# Reducing Admission for Anaphylaxis in a Pediatric Emergency Department Using a Clinical Decision Support Tool

**DOI:** 10.1097/pq9.0000000000000590

**Published:** 2022-09-08

**Authors:** Katherine H. Wolpert, Rebecca Kestle, Nicholas Weaver, Kelly Huynh, Minkyoung Yoo, Richard Nelson, Roni D. Lane

**Affiliations:** From the * Division of Emergency Medicine, Department of Pediatrics, University of Washington School of Medicine, Seattle, Wash.; † Division of Pediatric Emergency Medicine, Department of Pediatrics, Salt Lake City, Utah; ‡ Department of Pediatrics, Salt Lake City, Utah; § Department of Economics, University of Utah, Salt Lake City, Utah; ¶ Division of Epidemiology, University of Utah, Salt Lake City, Utah; ∥ Division of Pediatric Emergency Medicine, Department of Pediatrics, University of Utah School of Medicine, Salt Lake City, Utah.

## Abstract

**Introduction:**

Anaphylaxis is a life-threatening condition necessitating emergent management. However, the benefits of prolonged observation and indications for hospitalization are not well established. Through the implementation of a disposition-focused clinical decision support tool (CDST), this quality improvement initiative aimed to reduce hospitalization for low-risk patients presenting to the pediatric emergency department (PED) with anaphylaxis from 49% to ≤12% within 12 months of implementation.

**Methods:**

The intervention included patients 18 years and younger of age presenting with anaphylaxis to the PED. A multidisciplinary team identified a 2006 evidence-based guideline as a significant contributor to hospitalization. The updated guideline incorporated a disposition-focused CDST that stratified patients as low-risk or high-risk and recommended discharge of low-risk patients after a 4-hour observation period. The primary outcome measure was the percentage of low-risk patients hospitalized. Balancing measures included low-risk patient 72-hour return rate and PED length of stay for all comers. Secondary outcomes included a focused cost analysis.

**Results:**

Fifty-three children preintervention and 43 children postintervention presenting with anaphylaxis met low-risk criteria. Postimplementation, hospitalization of low-risk patients decreased from 49% to 7% (*P* < 0.0001). No low-risk patients returned in 72 hours for an anaphylaxis-related concern (*P* = 0.83). The median PED length of stay increased from 189 to 193 minutes (*P* < 0.0001). The median cost per low-risk encounter decreased by $377 (*P* = 0.013).

**Conclusions:**

After implementing an evidence-based disposition-focused CDST, hospitalization of low-risk patients presenting to the PED with anaphylaxis significantly decreased without an increase in 72-hour returns. In addition, patient encounters demonstrated cost savings.

## INTRODUCTION

Pediatric emergency department (PED) encounters for anaphylaxis are increasingly common in the United States (US).^1–3^ From 2011 to 2012, direct costs for pediatric food-allergy and anaphylaxis-related emergency department visits in the US approached $764 million annually, with inpatient stays costing $1.8 billion.^4^ In the acute care setting, prompt treatment of anaphylaxis with intramuscular (IM) epinephrine is the mainstay of treatment; however, the observation period following treatment in pediatric patients has not been standardized.^1,5,6^ Benefits of prolonged observation are unclear, and hospitalization rates vary widely.^1,5–9^ In 2014, a joint task force representing the American Academy of Allergy, Asthma, and Immunology; American College of Allergy, Asthma and Immunology, and the Joint Council of Allergy and Immunology published practice parameters recommending 4−8 hours of emergency department observation for patients without risk factors for severe anaphylaxis (eg, asthma, history of biphasic reaction, and protracted anaphylaxis).^6^

In a study of 35 PEDs between 2009 and 2013, hospitalization occurred in 41% of children who presented with anaphylaxis.^1^ Since then, some centers have reported reduced hospitalization rates by implementing care guidelines emphasizing PED discharge following a 4-hour observation period.^7,10,11^ Locally, baseline hospitalization of low-risk patients was 49%, and a local care guideline recommended admission for extended observation for patients presenting with anaphylaxis. This quality improvement (QI) initiative aimed to decrease hospitalization of low-risk patients from 49% to ≤12% in 12 months following the implementation of an updated evidence-based guideline (EBG) that incorporated a clinical decision support tool (CDST) to facilitate and standardize timely PED disposition.

## METHODS

### Context

The setting is a PED in a 289-bed quaternary care, university-affiliated, free-standing pediatric hospital. The PED has approximately 41,000 annual visits and an average admission rate of 19%. The children’s hospital is part of a large healthcare network comprised of 24 hospitals, 28 urgent care locations, and 215 clinics. In addition, the hospital serves as a referral center for the state and regions of four surrounding states. The hospital is at 4200 feet elevation and defines hypoxia as <90% room air saturation while awake (<88% while asleep). PED staffing includes pharmacists, nurses, technicians, pediatric emergency medicine board-certified physicians, pediatric emergency medicine fellows, general pediatricians, and advanced practice providers (APPs). Trainees in the PED included pediatric, family medicine, and emergency medicine residents. Trainees undergo orientation with an introduction to PED guidelines before their rotation.

For over 15 years, the PED has utilized evidence-based clinical decision support guidelines/CDSTs with a strong culture of buy-in by faculty and staff. The guidelines are descriptive, delineate role-specific tasks, utilize algorithms, and link with order sets. The guidelines and order sets were paper-based and housed in multiple PED locations before incorporation into the electronic health record (EHR) in October 2017. These items typically undergo regular reviews to ensure consistency of best practices and evidence-based literature.

In 2006, our institution implemented a PED anaphylaxis management guideline that recommended 12−24 hours of observation for all patients presenting to the PED with anaphylaxis. Prolonged observation with hospital admission of 49% of low-risk patients represented a quality gap and improvement opportunity. Patients presenting with anaphylaxis comprised <0.2% of the total PED census during the study period.

### Intervention

A multidisciplinary team of key stakeholders, including PED physicians, an APP, hospitalist, pharmacist, nurse, and nurse quality educator, formed a committee to evaluate the PED’s anaphylaxis practices. A pediatric allergist was available for consultation. The team performed a literature review and identified gaps in local versus current best practices involving the disposition of patients assessed as low risk. SQUIRE 2.0 guidelines outlined intervention, planning, and analysis.^12^

### Updated Evidence-based Guideline and CDST

**Fig. 1.**
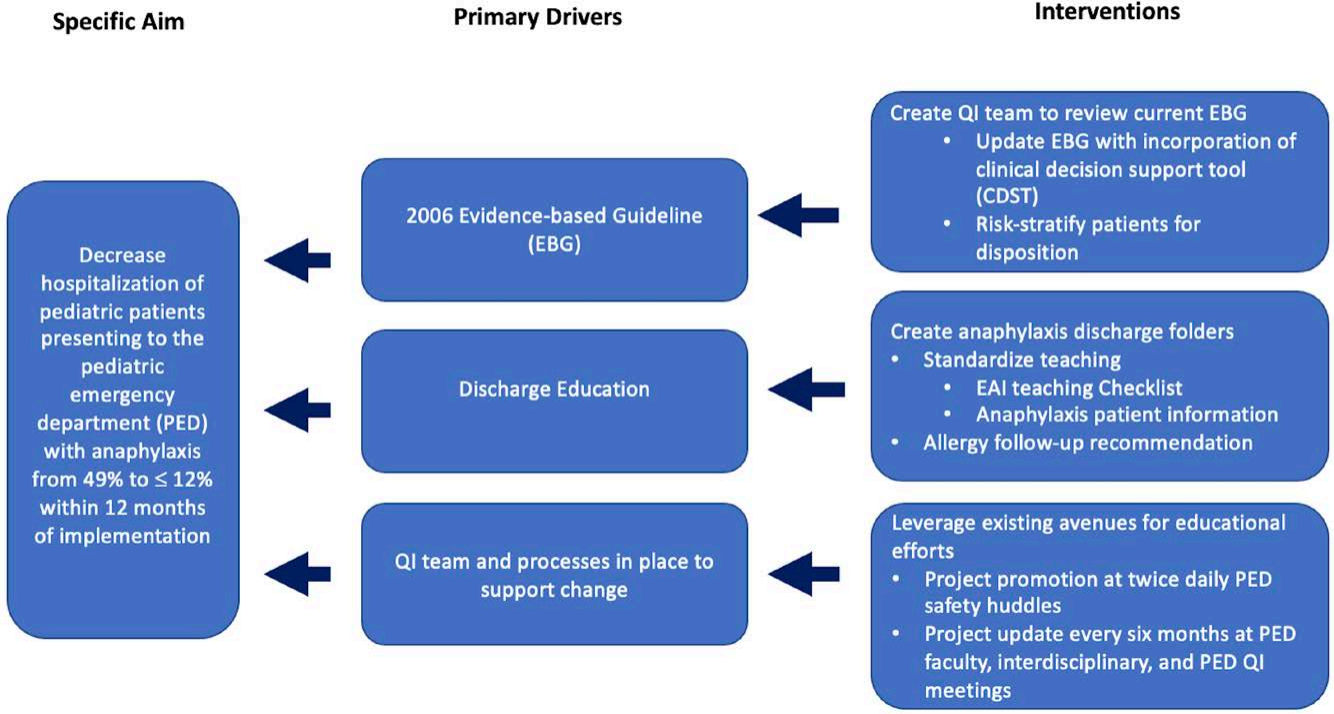
Key driver diagram.

**Fig. 2.**
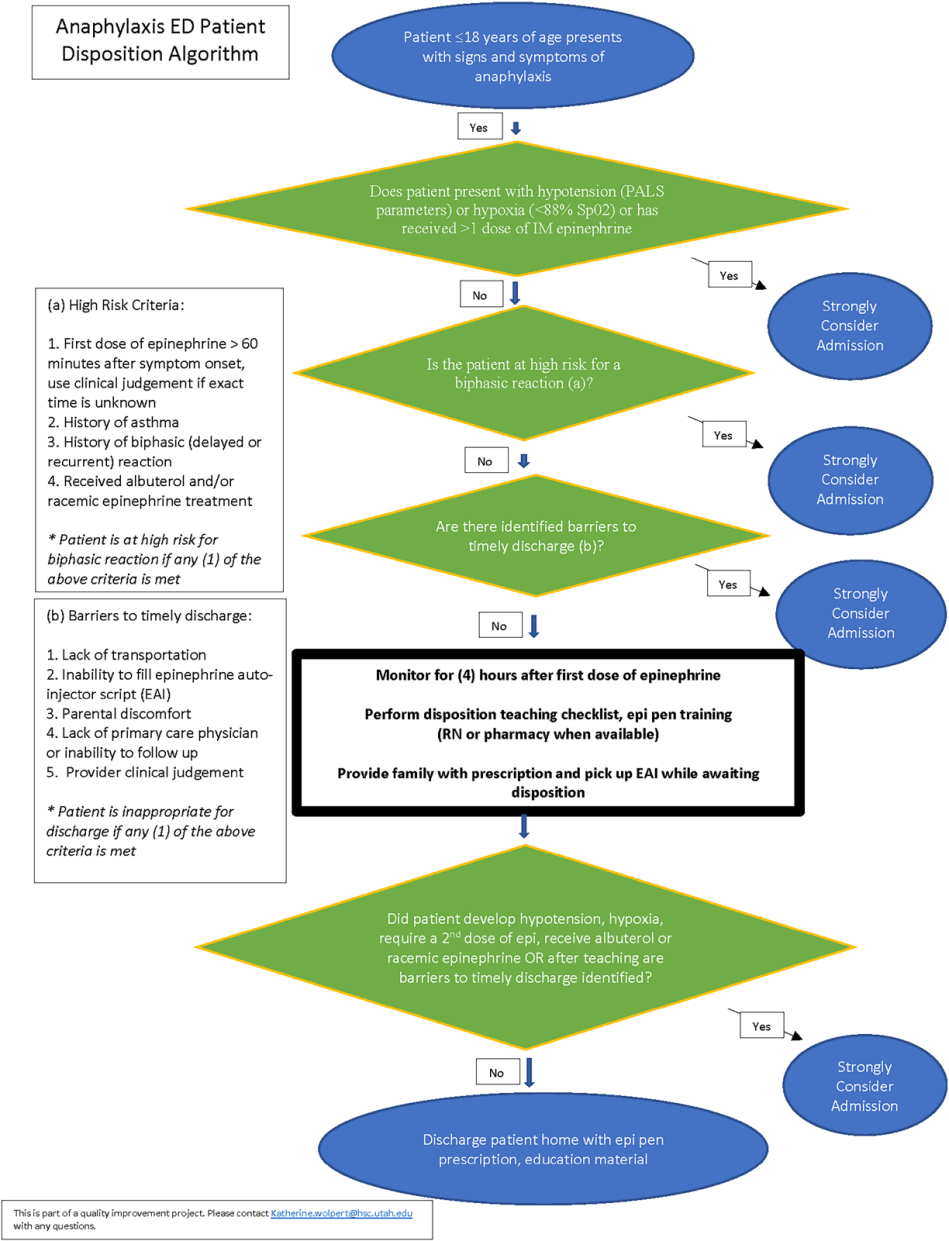
CDST. ED, emergency department; Epi, epinephrine; PALS, pediatric advanced life support; RN, registered nurse.

The institution’s 2006 anaphylaxis EBG highlighted triage, medical team response, interventions, and admission recommendation for extended observation. The multidisciplinary team created a key driver diagram (Fig. 1), which included updating the EBG with evidence-based best practices and incorporating a disposition-focused CDST (Fig. 2). The CDST recommends PED discharge for low-risk patients after a 4-hour observation period.

In the updated guideline, patients met low-risk criteria if they had no history of asthma, no history of biphasic reaction (recurrence of anaphylaxis within 72 hours of an initial reaction), received a prompt dose of IM epinephrine (within 60 minutes of symptom onset), did not demonstrate hypotension or hypoxia, and did not require albuterol, racemic epinephrine, or >1 dose of IM epinephrine.^8,9,13–17^ In addition to historical and clinical variables, risk stratification included social barriers. In addition, patients were at low risk if they had reliable transportation and timely outpatient follow-up, and could obtain an epinephrine autoinjector (EAI) at/before the time of discharge, and if the family felt comfortable administering IM epinephrine.

### Medical Staff and Patient Education

Over 6 months, team members presented drafts of the CDST at PED clinical management, nursing leadership, division faculty, and patient quality and safety council meetings, with opportunities for feedback and revision. Stakeholders presented the CDST and educational materials at their respective clinical staff meetings. PED physicians, APPs, pharmacists, and nursing leadership received the finalized version of the CDST via email. At the time of implementation, the team placed copies of the CDST throughout the PED for easy reference.

The multidisciplinary team also created anaphylaxis discharge folders to promote safe and efficient dispositions. Discharge folders contained information highlighting disease pathology, medication instructions, and return-to-care precautions for various common pediatric illnesses. In addition, the anaphylaxis discharge folders included a discharge checklist, educational anaphylaxis hand-outs, standardized EAI teaching materials, and an EAI teaching checklist. EAI prescriptions were included as a component of the discharge checklist, and families filled prescriptions before discharge. Education included the use of EAI trainers.

### Intervention Implementation

We implemented the CDST on July 1, 2018. The preintervention period was November 1, 2016–June 30, 2018. The postintervention period was July 1, 2018–December 31, 2019. Inclusion criteria were patients 18 years and younger of age who were diagnosed in the PED with an International Classification of Diseases, Ninth Revision, Clinical Modification (995.6−995.69 and 999.41−999.49) or International Classification of Diseases, Tenth Revision (ICD-10), Clinical Modification (T78.0-T78.09) code for anaphylaxis. (see Table, Supplemental Digital Content 1, http://links.lww.com/PQ9/A399, which shows ICD Codes with Description.) Also, the analysis included patients who received IM epinephrine for anaphylaxis before arrival (by a caregiver and/or prehospital provider) or during their PED stay. Exclusion criteria included patients who required intensive care unit admission or those who developed anaphylaxis due to an intervention in the PED after presenting a concern unrelated to anaphylaxis (eg, anaphylaxis related to contrast for an imaging study).

### Data Analysis

Data abstracted from the EHR and manual chart review included patient demographics, vital signs, medications administered, mode of arrival, patient origin (eg, home and clinic), PED disposition, and 72-hour returns. Pediatric Advanced Life Support parameters defined hypotension.^18^ Time to epinephrine administration was also measured. Epinephrine administration within 60 minutes of anaphylaxis symptom onset (time zero) defined timely administration. Emergency medical service records and caregiver’s report provided the time of epinephrine administration and symptom onset for patients whose time zero occurred before PED arrival. Timely epinephrine administration adjustment for patients whose time zero was estimated in the chart allowed an additional 30 minutes to account for recall inaccuracies. For example, if the PED note stated, “Symptoms started around 5:00 pm,” epinephrine given by 6:30 pm was considered timely. The CDST allowed for clinical judgment in cases where the time of symptom onset was unclear. Patients were included in the low-risk subset in these instances if the provider documented that the patient met low-risk criteria. Data collection and analysis occurred every 2 months.

### Study of the Intervention

**Fig. 3.**
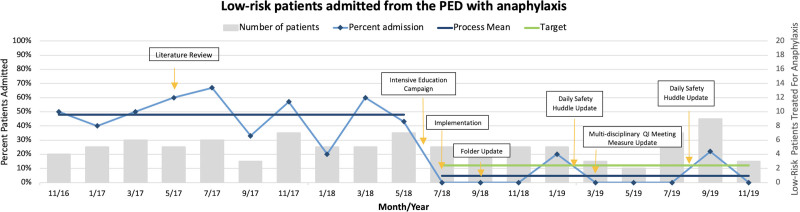
Run chart of low-risk patients admitted from the PED with anaphylaxis. Annotated run chart demonstrating a decrease in admission over the postimplementation period. Annotations are outlined.

We identified education and process improvement opportunities after implementation and initial data analysis. The monthly PED staff-wide newsletter and multidisciplinary PED twice-daily safety huddles highlighted project updates during the educational campaigns. Project education during safety huddles occurred over 1 week at 6-month intervals (Fig. 3). Furthermore, updates at the division’s multidisciplinary quality meetings provided additional education and a review of metric adherence.

### Early Disposition Teaching

Disposition teaching was traditionally done at the time of discharge. However, front-line nurses identified that performing discharge folder teaching and completing the discharge checklist earlier in the observation period allowed adequate time/resources for filling EAI prescriptions and helped identify barriers to successful discharge (eg, transportation, family comfort level, and lack of pediatrician follow-up). Moreover, during the observation period, completing discharge folder teaching also allowed for a more thorough review of anaphylaxis education materials, including a newly added patient-centered QR code. The code, which provided families with a resource to create an anaphylaxis action plan to take to their child’s school or activities, could be completed during the observation period.

### Early Epinephrine Auto-injector Prescription Filling

Changes to outpatient pharmacy hours prevented some families from filling their EAI prescriptions before discharge during the study period. To facilitate timely EAI dispensing, the PED team provided families with a list of 24-hour pharmacies, many within a 5−10 mile radius of the PED. This information encouraged caregivers to fill their prescriptions during the observation period or immediately after discharge at one of these locations.

### Measures

#### Primary Outcome

Our primary outcome was the percentage of patients with International Classification of Diseases, Ninth Revision or International Classification of Diseases, Tenth Revision codes for anaphylaxis admitted from the PED with anaphylaxis.

#### Secondary Outcome

Our secondary outcome was cost from the hospital perspective. We constructed this cost variable by summing the PED and inpatient costs for each encounter for all low-risk patients.

#### Balancing Measures

To determine whether the intervention increased anaphylaxis-related return visits, we included a balancing measure of a count/percent of any anaphylaxis-related return visit within 72 hours to any facility within the healthcare network. Return visits outside the network may not have been captured; however, the healthcare system represents the largest in the state and includes the only regional pediatric hospital. Moreover, to assess the potential impact of the intervention on the total PED population, we examined the overall PED length of stay (LOS).

### Analysis

Descriptive statistics included patient demographics and outcomes, including mean with standard deviation for continuous variables and counts and percentages for categorical variables. Descriptive statistics compared preperiods and postperiods. Chi-square tests assessed univariate comparisons of categorical variables, and Student *t* tests assessed continuous variables. Wilcoxson rank sum tests analyzed cost data. All statistical tests were evaluated at a two-sided alpha = 0.05.

Analyses were performed using SAS/STAT9.4 (SAS Institute Inc., Cary, NC, USA) and STATA 16 (StataCorp 2019, Stata Statistical Software: Release 16. College Station, TX: StataCorp LLC). In addition, an annotated run chart,^19,20^ created utilizing Microsoft Excel (Version 16.46.2016), evaluated the impact on low-risk patient admission.

### Ethical Considerations

The Institutional Review Board reviewed and approved this project. There were no financial relationships to disclose or conflicts of interest to resolve.

## RESULTS

Of the children presenting to the PED with anaphylaxis, 53 children preintervention and 43 children postintervention met low-risk criteria. We excluded one patient due to concurrent presentation of fever, lateral neck infection, and anaphylaxis. There were no differences in age, gender, mode of arrival to the PED, place of origin, or adjunctive medications received between the preimplementation and postimplementation low-risk groups (Table 1).

**Table 1.** Characteristics of Low-risk Patients Presenting to the PED with Anaphylaxis

Characteristic	Preintervention(n = 53)	Postintervention (n = 43)	*P*
Age in Years (SD)	7.0 (5.5)	6.6 (5.3)	0.735
Male gender, n (%)	29 (55)	20 (47)	0.424
White, n (%)	44 (83)	30 (70)	0.283
Ethnicity, n (%)			
Hispanic	9 (17)	11 (26)	0.408
Non-Hispanic	43 (81)	32 (74)	
Missing	1 (2)	0 (0)	
Mode of PED arrival, n (%)	34 (64)	26 (61)	0.642
Personal Vehicle	19 (36)	16 (37)	
EMS	0 (0)	1 (2)	
Other			
Patient origin, n (%)			
Home	38 (72)	29 (67)	0.523
Clinic/urgent care	11 (21)	12 (28)	
Referral hospital	4 (8)	2 (5)	
IM epinephrine, n (%)			
In PED	20 (38)	14 (33)	0.598
Before arrival	33 (62)	29 (67)	
Steroid, n (%)			
In PED	41 (77)	35 (81)	0.399
Before arrival	8 (15)	3 (7)	
Antihistamine, n (%)			
In PED	40 (76)	35 (81)	0.747
Before arrival	4 (8)	3 (7)	
Diphenhydramine, n (%)			
In PED	23 (43)	21 (49)	0.612
Before arrival	28 (53)	19 (44)	

Categorical data were evaluated using Chi-square analysis, and continuous data presented as mean using the Student *t* test.

EMS, emergency medical services; SD, standard deviation.

Postimplementation, the mean percent of low-risk patient admissions decreased from 49% to 7% (*P* < 0.0001) (Table 2), demonstrating improvement over time (Fig. 3). This finding confers an absolute risk reduction of 42% and a relative risk reduction of 86% in the admission of low-risk patients. Mean values were chosen (over median) given the number of periods with zero admissions. Postimplementation, providers admitted three children (7%), despite qualifying as low risk per CDST recommendations. Providers noted discomfort with age (two patients younger than 12 months) and prehospital vital signs (concern for hypotension) as the primary cause of hospitalization.

**Table 2.** Outcome Analysis Preintervention and Postintervention

Outcome	Preintervention	Postintervention	*P*
PED disposition, n (%)			
Home	27 (51)	40 (93)	
Admission	26 (49)	3 (7)	<0.001
PED low-risk LOS min (mean, SD)	190 (58)	232 (47)	<0.002
PED all-comers LOS min (median, SD)	189 (9)	193 (6)	<0.001
Total cost, dollars (median, IQR)	826 (1064)	449 (483)	0.013
72-h return visits, n (%)			
Anaphylaxis-related	0 (0)	0 (0)	0.831
Scheduled follow-up or unrelated to anaphylaxis	2 (4)	2 (5)	

Categorical data evaluated using Chi-square analysis, and continuous data presented as mean using Student *t* test. Cost has been evaluated using Wilcoxon rank sum test.

LR, low risk.

Table 2 describes additional outcomes and results. In the preimplementation and postimplementation low-risk groups, no patients returned in 72 hours for anaphylaxis-related concerns (*P* = 0.83). The median cost per low-risk patient encounter decreased postimplementation from $826 to $449 (*P* = 0.013). The mean PED LOS for low-risk patients increased from 190 to 232 minutes postintervention (*P* < 0.0002); however, the median LOS for all PED patients increased marginally from 189 minutes preintervention to 193 minutes postintervention (*P* < 0.0001).

## DISCUSSION

By revising the preexisting EBG and incorporating a disposition-focused CDST, we demonstrated a relative reduction of 86% (from 49% to 7%) in hospital admission among low-risk patients treated for anaphylaxis in a PED, surpassing our goal. To our knowledge, this report is the first study describing the impact of a CDST on the low-risk pediatric patient population. Project success was multifactorial, including early, extensive CDST adoption and a preexisting EBG acceptance and adherence culture.

Hospitalization of pediatric patients presenting with anaphylaxis is significant. In a 2016 study of 35 PEDs, Michelson et al reported hospitalization in 41% of children presenting with anaphylaxis.^1^ Since then, a few centers have published work on decreasing hospitalization among this population.^7,10,11^ Risk stratification criteria vary among studies. This study differs by including the time-to-first dose of epinephrine from symptom onset in risk stratification. The 2020 GRADE analysis supports this strategy, in which 3 of 8 studies demonstrated a significantly increased risk of biphasic reaction with delayed IM epinephrine administration. A fourth study noted a protective effect against biphasic reaction after administering IM epinephrine within 90 minutes of symptom onset.^21^

PED anaphylaxis management incurs high costs for emergency department and inpatient care.^4,22^ A 2011 cost analysis by Patel et al^22^ reported that the average direct emergency department costs, including facility and physician charges, were $711 per patient presenting to the ED with anaphylaxis, of whom 38% were children. Our study demonstrated average facility charges (PED plus inpatient stay) of $826 in the low-risk patient preintervention group, which decreased to $449 in the postintervention group, likely reflecting fewer patients being admitted. Although we did not include physician charges and did not separate unit-specific costs, our study demonstrated an overall facility cost reduction of 46% in low-risk patients. Regarding the balancing measures, we demonstrated no increase in anaphylaxis-related 72-hour returns in low-risk patients discharged from the PED, comparable to previous studies.^7,10^ There was also a marginal increase in the median LOS of 4 minutes for all PED patients (189–193 minutes). Although statistically significant, this is unlikely clinically significant. Patients with anaphylaxis comprise a very small portion of the overall PED population (<0.2%). Other factors may have influenced overall LOS, including other initiatives occurring simultaneously in the PED and unknown contextual factors. As anticipated, PED LOS increased among low-risk patients preintervention and postintervention (190 and 232 minutes, respectively), similar to findings by Farbman et al.^7^

This QI initiative required PED resources. The 4-hour observation period required additional monitoring, nursing time, anaphylaxis, and EAI education and teaching. These factors may have affected the availability of personnel to address other tasks within the PED, potentially impacting patient wait times or delaying clinical care. We attempted to mitigate this by incorporating teaching and educational tasks into existing workflows during the observation period rather than at discharge time. Though not measured directly, we suspect that performing EAI teaching and discharging with EAI in hand are value-added steps in patient care.

### Limitations

This study is not without limitations. Given the retrospective study design, data were limited to what was available in the EHR. To enhance data integrity, we performed manual chart reviews. Allowing an additional 30 minutes from symptom onset to epinephrine administration from the estimated time-zero likely increased the denominator of low-risk patients, which may have influenced the impact of the intervention. Due to inconsistent documentation in the EHR, the analysis excluded interventions, other than IM epinephrine, given before hospital arrival. This strategy could have resulted in inappropriately assigning patients to the low-risk cohort. However, applying the same strategy to the preimplementation and postimplementation cohorts reduced potential selection bias. The cost analysis included only facility charges. Including physician charges may have impacted the conclusions by increasing costs.

Additionally, the cost analysis did not include indirect costs such as missed work, the impact of stressful hospitalization, or other social factors. No patient returned in 72 hours for an anaphylaxis-related concern during the study period, which is likely a reflection of the small number of study participants, and may have limited the power and ability to demonstrate statistical significance. Finally, this single-center study has a longstanding EBG adoption and acceptance culture. These factors may limit generalizability, but we are hopeful that adapting the intervention strategies and CDST to meet the needs of different centers allows widespread use.

### Future Considerations

Recommendations from the GRADE 2020 analysis, published after completing this project, provide guidance for future improvements. It recommends discharging patients without severe anaphylaxis after a 1-hour observation period, offering further opportunities for decreased LOS and lower patient costs.^21^ We anticipate conducting a plan-do-study-act cycle incorporating these recommendations, which will involve education and amending the CDST.

Additionally, applying these new recommendations may broaden the definition of low risk and further reduce admission and cost. Although the variation of risk stratification standards makes study comparisons challenging, risk classifications, definitions, and approaches to disposition must be nimble as new evidence emerges.

## CONCLUSION

After implementing a disposition-focused CDST, hospitalization of low-risk patients presenting to the PED with anaphylaxis significantly decreased without an increase in 72-hour returns. Additionally, it was associated with a lower cost per encounter.

## ACKNOWLEDGMENTS

Assistance with study: We thank Nicholas Weaver for his expertise and participation in the pharmacist role of this project and Rebecca Kestle for her involvement in promoting the project and facilitating communication with multidisciplinary staff. We are grateful to Dr. Howard Kadish for his guidance and support of this project in the pediatric emergency department.

## DISCLOSURE

The authors have no financial interest to declare in relation to the content of this article.

## Supplementary Material


