# Supplementary material for: Reducing Admission for Anaphylaxis in a Pediatric Emergency Department Using a Clinical Decision Support Tool
Source: Pediatr Qual Saf. 2022 Sep 8;7(5):e590. doi: 10.1097/pq9.0000000000000590 (PMC10997234; doi:10.1097/pq9.0000000000000590)
Supplement: Supplementary file 1 [file pqs-7-e590-s001.pdf]

Supplemental Table 1. ICD Codes with Description		
ICD Code	Version	Description
995.6	9	ANPHYL RCTN FOOD NOS
995.61	9	ANPHYL RCTN PEANUTS
995.62	9	ANPHYL RCTN CRUSTACEANS
995.63	9	ANPHYL RCTN FRUIT/VEGGIE
995.64	9	ANPHYL RCTN TREENUT/SEED
995.65	9	ANPHYL RCTN DUE TO FISH
995.66	9	ANPHYL RCTN FOOD ADDITIV
995.67	9	ANPHYL RCTN MILK PRODUCT
995.68	9	ANPHYL RCTN DUE TO EGGS
995.69	9	ANPHYL RCTN FOOD NEC
999.41	9	ANPHYL RCTN 2ND ADM BLD
999.42	9	ANPHYL RCTN 2ND VACCINE
999.49	9	ANPHYL RCTN 2ND SRUM NEC
T78.00	10	Anaphylactic reaction due to unspecified food
T78.01	10	Anaphylactic reaction due to peanuts
T78.02	10	Anaphylactic reaction due to shellfish (crustaceans)
T78.03	10	Anaphylactic reaction due to other fish
T78.04	10	Anaphylactic reaction due to fruits and vegetables
T78.05	10	Anaphylactic reaction due to tree nuts and seeds
T78.06	10	Anaphylactic reaction due to food additives
T78.07	10	Anaphylactic reaction due to milk and dairy products
T78.08	10	Anaphylactic reaction due to eggs
T78.09	10	Anaphylactic reaction due to other food products
ICD, International Classification of Disease		
